# LC–MS-based serum metabolomics reveals distinct metabolic signatures in patients with cerebral infarction

**DOI:** 10.3389/fneur.2026.1714257

**Published:** 2026-04-09

**Authors:** Dayong Guo, Jingya Li, Huaiyong Yang, Xiaohui Lu

**Affiliations:** People's Hospital of Bayingol Mongolian Autonomous Prefecture, Kuerle, China

**Keywords:** cerebral infarction, LC–MS, metabolites, metabolome, serum

## Abstract

Cerebral infarction is a common cerebrovascular disease associated with high mortality. This study compared the metabolic profiles and class distributions between patients with cerebral infarction (*n* = 10) and a control group without cerebral infarction (*n* = 10) using serum-based metabolomics. A total of 3,160 metabolites were identified in the serum of patients with cerebral infarction, among which benzene and its substituted derivatives accounted for the largest proportion (15.71%), followed by amino acids and their metabolites (12.39%), organic acids and their derivatives (12.32%), and heterocyclic compounds (12.05%). In the control group, amino acids and their derivatives accounted for the largest proportion (18.49%). Principal component analysis (PCA) and OPLS-DA models revealed clear metabolic separation between the two groups, indicating pronounced metabolic reprogramming in patients with cerebral infarction. A total of 329 differential metabolites were identified between patients with cerebral infarction (*n* = 10) and control subjects. Serum levels of benzene derivatives, organic acids and bile acids were significantly elevated in patients with cerebral infarction, while levels of amino acids, their derivatives and glycerophospholipids were reduced. Network analysis revealed strong positive correlations among differentially expressed lipid metabolites, and benzene derivatives showed negative correlations with amino acid-related metabolites. Enrichment analysis also revealed that these differentially expressed metabolites were primarily involved in lipid metabolism, amino acid metabolism, energy metabolism and inflammation-related pathways. The study demonstrated significant abnormalities in the serum metabolic profiles of patients with cerebral infarction, characterized by disturbances in aromatic compound and lipid metabolism. This provides a basis for the screening of potential biomarkers and the study of pathological mechanisms of cerebral infarction.

## Introduction

Cerebral infarction is an acute cerebrovascular disease caused by impaired cerebral blood circulation, resulting in localized brain tissue ischemia, hypoxic necrosis or softening (1, 2). Cerebral infarction accounts for 70–80% of all strokes and is the most common type of stroke. High mortality, high disability rates, and a high risk of recurrence are the main clinical features of cerebral infarction (3). Most patients experience significant neurological deficits in the acute phase, while survivors often suffer from varying degrees of sequelae, such as hemiplegia, aphasia, and cognitive impairment, which severely impair their quality of life (4). Furthermore, cerebral infarction has a high tendency to recur that makes it a significant cause of long-term disability and mortality.

The complex pathological mechanisms of cerebral infarction, the narrow treatment time window, the high risk of complications and non-regenerative neurons are the main factors contributing to the difficulty of treatment (5). The optimal time for treatment is within 4.5 h after the onset of cerebral infarction. Administering treatment beyond this time window reduces the effectiveness of thrombolysis and may result in irreversible necrosis of ischemic brain tissue, which can increases the difficulty and effectiveness of the treatment (6). In addition, about 16–40% of patients with acute cerebral infarction experience worsening neurological deficits within 48–72 h that further shortens the effective treatment time. In clinical practice, the FAST principle of rapid identification and imaging examinations are the main methods for identifying patients with cerebral infarction (7).

Inflammatory factors (8), metabolite profiling (9) and circulating RNA biomarkers (10) are being explored as aids in the early diagnosis of diseases. The high sensitivity and reproducibility of metabolites, along with their ability to reflect early metabolic abnormalities, are the primary reasons for their use in clinical diagnostics. Metabolite profiling has been widely used for detecting a variety of diseases, including cardiovascular disease, diabetes, cancer, neurological disorders and liver and kidney dysfunctions (11–13). A study used machine learning to analyze a gastric cancer diagnostic model containing 10 metabolites and identified two distinct biomarker panels for early detection and prognosis prediction (14). A study conducted a metabolome-wide association study (MWAS) of Parkinson’s disease (PD) patients and non-PD patients found metabolites associated with Parkinson’s disease, such as phenylacetyl-L-glutamine, trigonelline, biliverdin and pantothenic acid (15).

Currently, systematic studies of the serum metabolome in patients with cerebral infarction are limited. In this study, we used liquid chromatography–mass spectrometry (LC–MS) to comprehensively analyze the serum metabolome and its compositional characteristics in 10 patients with cerebral infarction and 10 healthy controls. This study identified characteristic metabolic alterations and potential biomarkers associated with cerebral infarction. These findings not only contribute to understanding the molecular metabolic mechanisms underlying cerebral infarction but also provide important insights for early diagnosis, disease evaluation, and targeted intervention.

## Materials and methods

### Participants

This study enrolled 20 subjects from the People’s Hospital of Bayingol Mongolian Autonomous Prefecture, including 10 patients with clinically and radiographically confirmed cerebral infarction (C001–C010) and 10 healthy controls (D001–D010) (Supplementary Table S1). Patients with cerebral infarction were assigned to the experimental group, while individuals without cerebral infarction were included in the control group.

Inclusion criteria were: (1) Age between 18 and 70 years. (2) Cerebral infarction confirmed by clinical evaluation and radiographic examination. All patients were independently assessed and diagnosed by two neurologists with relevant expertise. Clinical diagnostic criteria included the presence of focal neurological deficits with acute or subacute onset lasting more than 24 h, such as hemiplegia, speech impairment, sensory abnormalities, visual field defects or ataxia. Radiographic diagnosis was primarily based on cranial magnetic resonance imaging (MRI), with diffusion-weighted imaging (DWI) demonstrating high-signal lesions consistent with clinical symptoms and corresponding low-signal changes on apparent diffusion coefficient (ADC) maps. (3) All subjects voluntarily participated and provided written informed consent. Venous blood samples were collected for metabolomics analysis.

Exclusion criteria were (1) History of acute or chronic infection within 2 weeks prior to sampling. (2) Pregnant or lactating women. (3) Use of medications that may affect the metabolic profile (e.g., glucocorticoids, chemotherapy drugs) within 1 month prior to sampling. (4) Patients with severe liver, kidney, or heart failure or malignant tumors. (5) Patients with psychiatric illness or other conditions that may affect compliance and cooperation. The study protocol was approved by the Ethics Committee of the People’s Hospital of Bayingol Mongolian Autonomous Prefecture (Approval Number: BZRMYY-LCYJ-2024-50), and all participants provided written informed consent.

### Detection of metabolites

Metabolite extraction and LC–MS/MS analysis were performed according to the non-targeted metabolomics protocol published by Saigusa et al. (16). All subjects fasted overnight (≥8 h) before blood collection. 5 mL of peripheral blood was drawn by venipuncture into a vacutainer. After 30 min of clotting at room temperature, blood samples were centrifuged at 3,000 × g for 10 min at 4 °C. The resulting serum was transferred to nuclease- and protease-free 1.5 mL cryogenic tubes and immediately stored at −80 °C. Serum samples were slowly thawed on ice and gently mixed. For each 100 μL of serum, 300 μL of pre-chilled methanol/acetonitrile (20% v/v) was added and mixed thoroughly for 3 min to precipitate proteins. Samples were centrifuged at 12,000 × g for 10 min at 4 °C, and 200 μL of the supernatant was transferred to a fresh tube. After standing at −20 °C for 30 min, samples were centrifuged again under the same conditions for 3 min, and 180 μL of the clarified solution was collected and transferred to an LC–MS/MS injection vial.

Chromatographic separation was performed on a Shimadzu LC-30A UPLC system equipped with a Waters ACQUITY Premier HSS T3 column (2.1 × 100 mm, 1.8 μm). The mobile phase consisted of 0.1% formic acid in water (A) and 0.1% formic acid in acetonitrile (B) at a flow rate of 0.4 mL/min. The column temperature was maintained at 40 °C, and the injection volume was 4 μL. Gradient elution was performed as follows: 95% A/5% B at 0.0 min, 80% A/20% B at 2.0 min, 40% A/60% B at 5.0 min, 1% A/99% B at 6.0–7.5 min, returning to 95% A/5% B at 7.6–10.0 min.

Mass spectrometry was performed using an AB SCIEX TripleTOF 6,600 system (Foster City, USA) in both positive (ESI^+^) and negative (ESI^−^) ion modes. The total acquisition time was 10 min, including both MS1 and MS2 scans. The spray voltage was +5,000 V for ESI^+^ and −4,000 V for ESI^−^. Source temperatures were 550 °C for ESI^+^ and 450 °C for ESI^−^. Gas settings were as follows: nebulizer gas 50 psi, heater gas 60 psi, and curtain gas 35 psi. The declustering potential (DP) was ±60 V, and collision energy (CE) was ±10 V for MS1 and ±30 V for MS2 with an energy spread of 15 V. Mass scan ranges were m/z 50–1,000 for MS1 and m/z 25–1,000 for MS2. Acquisition rates were 0.2 s for MS1 and 0.04 s for MS2. Dynamic exclusion was set to select the first 18 precursor ions, triggered three times, followed by a 3 s exclusion period.

### Quality control

In this study, one quality control (QC) sample was included for every 10 serum samples to ensure the reliability of the analytical results. The quantitative data of all metabolites were used to calculate the coefficient of variation (CV), and cumulative distribution curves were plotted to assess the stability and reproducibility of the detection platform. After data normalization, principal component analysis (PCA) (17) was performed using R to explore metabolic differences and potential classification patterns between patients with cerebral infarction (group C) and healthy controls (group D) from an unsupervised perspective. Additionally, hierarchical cluster analysis (HCA) was conducted based on standardized metabolite abundances to compare overall metabolic expression patterns between the two groups.

### Orthogonal partial least squares discriminant analysis

Orthogonal partial least squares discriminant analysis (OPLS-DA) was applied to evaluate global metabolomic differences between patients with cerebral infarction and healthy controls. Metabolite abundances were log-transformed and normalized using the Pareto method. The discriminant model was established and visualized using the ropls package (18) in R. Model stability and reliability were assessed via 200 random permutation tests. Key differential metabolites were screened based on variable importance in projection (VIP) values, with VIP > 1.0 considered significant.

### Identification of differential metabolites

Metabolite annotation was performed by combining high-resolution MS data with publicly available metabolite databases. MS1 and MS2 spectra were compared with Human Metabolome Database (HMDB), considering accurate mass, isotopic pattern, adduct type and fragmentation pattern. The annotation process parameters include mass tolerance (±5 ppm for MS1, ±10 ppm for MS2), isotopic similarity threshold (≥80%), adduct type ([M + H]+, [M + Na] + and [M + K] + in positive ion mode, [M − H] − in negative ion mode), and fragment matching (minimum spectral similarity score of 0.7).

Statistical analyses of the normalized serum metabolomics data were conducted in R. Log₂ fold changes (log₂FC) and *p*-values of differential metabolites were calculated using the limma package (19), and the Benjamini–Hochberg method was applied to control the false discovery rate (FDR). For variables not meeting normality assumptions, non-parametric analyses were performed using the Wilcoxon rank-sum test. Differential metabolites were defined by the following criteria: VIP > 1.0, adjusted *p* < 0.05, and |log₂FC| > 1.0. Volcano plots were generated using ggplot2 (20), and metabolite classification and functional annotation were performed with MetaboAnalystR (21).

R software was used to perform statistical analysis on the processed and normalized serum metabolomics data of patients with and without cerebral infarction. The log₂(FC) and *p*-value of differential metabolites were calculated using the *limma* package (19), and the Benjamini–Hochberg method was used to correct for multiple hypothesis testing to control the FDR. For variables that did not meet the normal distribution assumption, the Wilcoxon rank sum test in the stats package was used for non-parametric analysis. Differential metabolites were defined by the following criteria: VIP > 1.0, adjusted *p* < 0.05, and |log₂FC| > 1.0. The results were visualized by drawing volcano plots using the *ggplot2* package (20), and metabolite classification and functional annotation were completed with the help of the *MetaboAnalystR* package (21).

### Enrichment analysis

Pathway enrichment analysis of differential metabolites was performed using the enricher() function in the *clusterProfiler* package (22) with the main parameters set to pvalueCutoff = 0.05, qvalueCutoff = 0.2, minGSSize = 10 and maxGSSize = 500. The analysis results were further linked to the org Hs.eg.db database via setReadable() to convert the KEGG IDs into readable metabolite/gene symbols.

A regulatory network was constructed based on the KEGG enrichment results and visualized using *igraph* (v1.5.1) (23) and MetaboAnalystR (v3.2.0) (21). Differential metabolites were mapped to HMDB (24), and metabolite set enrichment analysis (MSEA) was performed using the msetEnrichment() module in MetaboAnalystR. This analysis compared differential metabolites to disease-related metabolite sets to evaluate their enrichment trends under various pathological or physiological conditions, revealing potential associations with human metabolic diseases (parameters: pvalueCutoff = 0.05, FDR = TRUE).

## Results

### Serum metabolite profile characteristics and category distribution in patients with and without cerebral infarction

**Figure 1:**
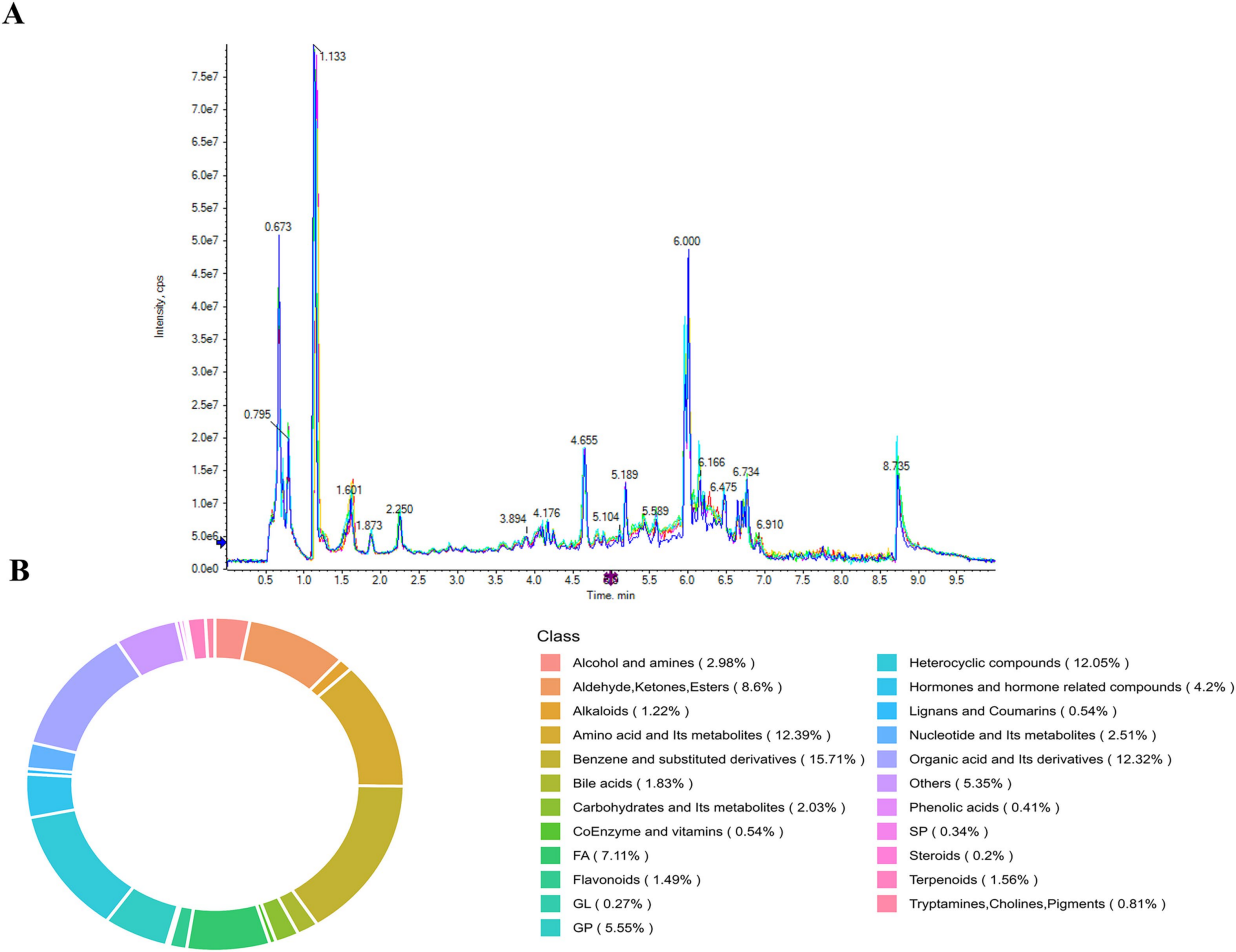
Serum metabolomic analysis of patients with cerebral infarction **(A)**. Total ion current chromatograms (TIC) of serum from patients with cerebral infarction **(B)**. Analysis of metabolite class proportions in serum from patients with cerebral infarction.

In this study, serum metabolomes were obtained from 10 patients with cerebral infarction and 10 healthy controls. The mean age of patients with cerebral infarction was 58.3 ± 9.46 years, and that of controls was 55.7 ± 11.08 years. TIC analysis showed multiple characteristic peaks in the serum of patients with cerebral infarction at retention times of 0.673, 0.795, 1.133, 1.601, 2.250, 4.655, 6.000, and 8.735 min (Figure 1A), while controls exhibited peaks at 0.654, 1.142, 1.653, 2.256, 5.997, and 8.732 min (Supplementary Figure S1A). A total of 3,160 metabolites were identified across all serum samples. Analysis of the compounds corresponding to characteristic peaks in patients with cerebral infarction showed that benzene and its substituted derivatives accounted for the largest proportion (15.71%) (Figure 1B), followed by amino acids and their metabolites (12.39%), organic acids and their derivatives (12.32%), heterocyclic compounds (12.05%) and fatty acids (7.11%). The highest concentrations of benzene derivatives suggest that abnormalities in aromatic metabolism may contribute to the pathogenesis of cerebral infarction. In healthy controls, amino acids and their metabolites were most abundant (18.49%), followed by benzene and substituted derivatives (14.41%), heterocyclic compounds (10.64%), glycerophospholipids (10.02%), and organic acids and their derivatives (9.09%) (Supplementary Figure S1B). These results demonstrate significant abnormalities in the serum metabolite profiles of patients with cerebral infarction, particularly the marked elevation of benzene and its derivatives.

### Data quality assessment of serum metabolomics

**Figure 2:**
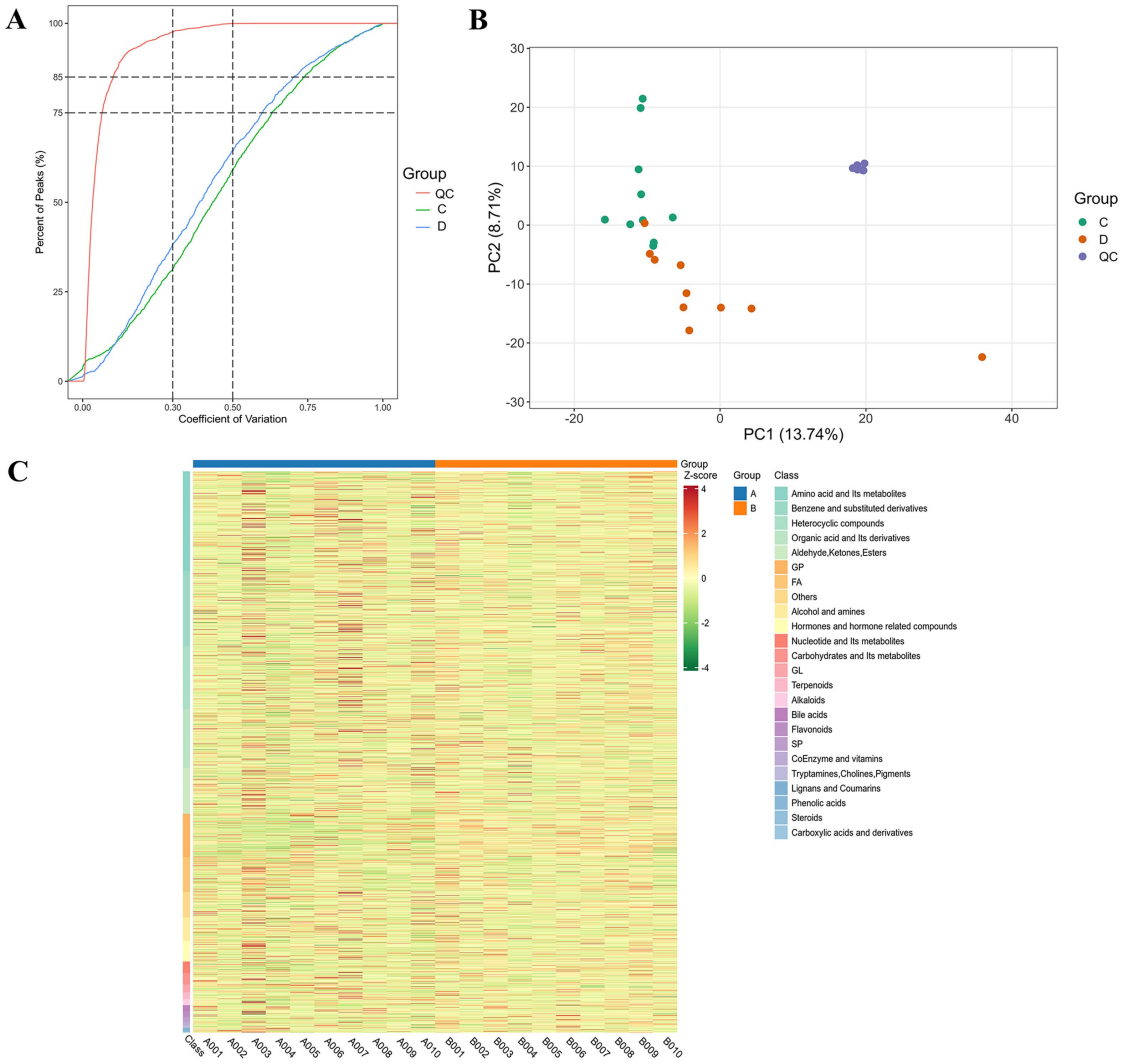
Stability and intergroup differences in serum metabolomics data between patients with and without cerebral infarction. **(A)** Cumulative distribution analysis of the coefficient of variation. **(B)** PCA analysis of serum metabolomics and intergroup differences between patients with and without cerebral infarction. **(C)** Cluster analysis of serum metabolite expression in patients with and without cerebral infarction. QC is quality control, A is the metabolome of patients with cerebral infarction, B is the metabolome of patients with and without cerebral infarction.

This study further evaluated the serum metabolomic profiles of patients with cerebral infarction using a multidimensional analysis system (Figure 2). Cumulative distribution analysis of the coefficient of variation (CV) (Figure 2A) showed that the CVs of metabolites in the control group were significantly lower than those in patients with cerebral infarction (CV < 0.3, explanation rate > 75%), indicating that the metabolomics data were high quality. The higher dispersion observed in patient samples (CV > 0.5, explanation rate > 30%) may reflect disease heterogeneity. Principal component analysis (PCA) (Figure 2B) revealed a clear separation of 13.74% along the PC1 axis between patients and controls, indicating significant metabolic reprogramming in cerebral infarction. The tight clustering of control samples confirmed the stability and reproducibility of the experiment. Differential metabolite heatmap analysis (Figure 2C) further revealed the specific enrichment of benzene derivatives and amino acid metabolites in patients with cerebral infarction (red blocks). These signature metabolites may serve as candidate biomarkers for cerebral infarction.

### Metabolomics analysis of OPLS-DA in patients with and without cerebral infarction

**Figure 3:**
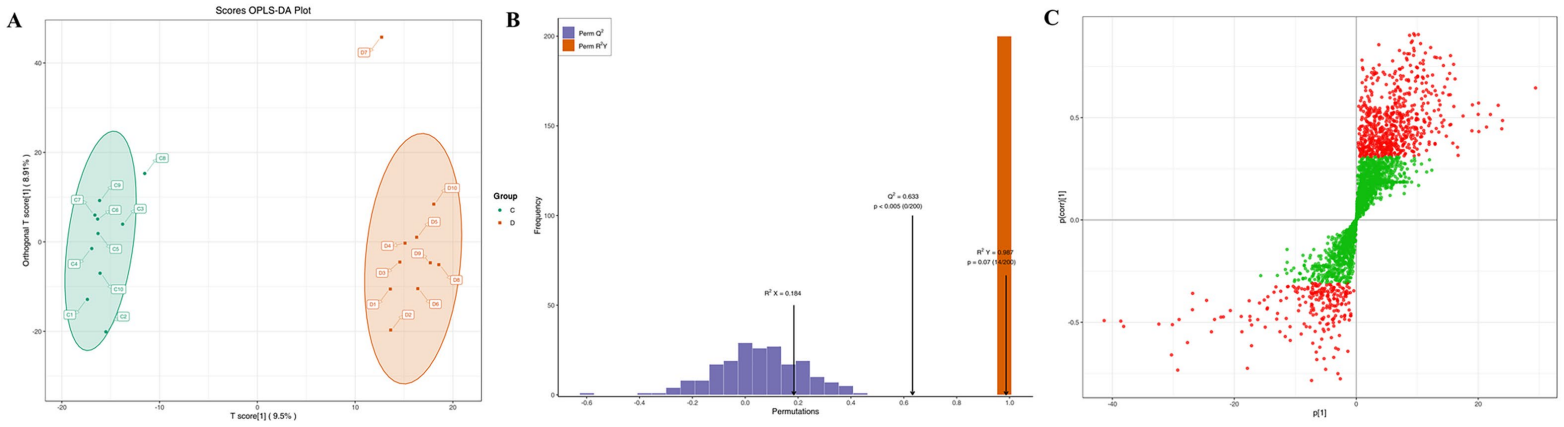
OPLS-DA model analysis of serum metabolomics in patients with and without cerebral infarction. **(A)** OPLS-DA score plot of the serum metabolome of patients in the cerebral infarction and non-cerebral infarction groups. **(B)** OPLS-DA permutation test of the serum metabolome of patients in the cerebral infarction and non-cerebral infarction groups. The horizontal axis represents the model R2Y and Q2 values, and the vertical axis represents the frequency of model classification effects in 200 random permutation experiments. **(C)** OPLS-DA loading scatter plot of the serum metabolome of patients in the cerebral infarction and non-cerebral infarction groups. Red dots indicate metabolites with a VIP value greater than 1, and green dots indicate metabolites with a VIP value less than or equal to 1.

The OPLS-DA model was used to compare the differences in serum metabolic profiles between patients with cerebral infarction (Group C) and patients without cerebral infarction (Group D) (Figure 3). The score plot (Figure 3A) showed that the two groups of samples were significantly separated along the axis (Group C on the left and Group D on the right), suggesting that there were significant differences in their metabolic characteristics (*p* < 0.01). The results of the permutation test (Figure 3B) showed that the R^2^Y (0.987) and Q^2^ (0.633) of the original model were significantly higher than those of the random model (*p* < 0.01), verifying the reliability of the model. VIP analysis (Figure 3C) further screened out key metabolites that contributed significantly to the distinction between the two groups (VIP > 1, marked in red). These metabolites were predominantly located in the right region of the score plot and were involved in pathways, such as oxidative stress and energy metabolism. The OPLS-DA results effectively distinguished the serum metabolomes of patients with cerebral infarction from those of controls, providing strong statistical support for the selection of candidate biomarkers.

### Differential metabolites in patients with and without cerebral infarction

**Figure 4:**
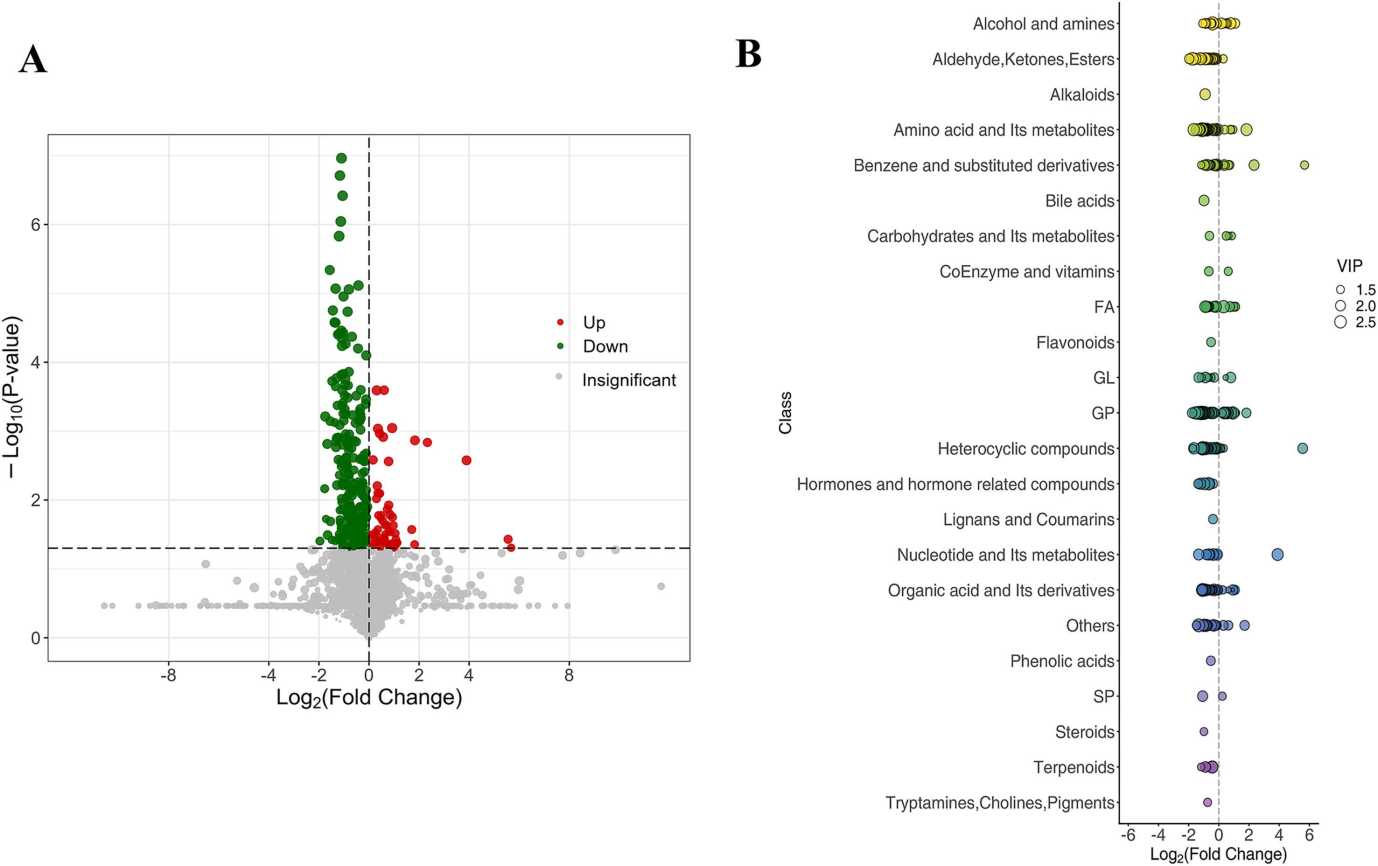
Differential metabolites in the serum metabolome of patients with and without cerebral infarction. **(A)** Volcano plot of differential metabolites in patients with and without cerebral infarction. **(B)** Classification of differential metabolites in patients with and without cerebral infarction.

We compared the serum metabolic profiles of patients with and without cerebral infarction to identify differentially expressed metabolites (Figure 4). A volcano plot (Figure 4A) revealed significant differences in 329 metabolites (Supplementary Table S2), of which 271 were significantly upregulated (red dots) and 58 were significantly downregulated (green dots) in the cerebral infarction group. The remaining 2,831 metabolites showed no significant differences (gray dots). Further analysis (Figure 4B) revealed that serum levels of benzene and its substituents, organic acids, and bile acids were significantly elevated in cerebral infarction patients compared with controls, while amino acids, their derivatives and glycerophospholipids (GPs) showed a downward trend. These results suggest significant metabolic reprogramming in cerebral infarction patients, particularly in the abnormalities of aromatic compounds and energy metabolism pathways. In contrast, flavonoids and vitamins/coenzymes did not exhibit significant differences between the two groups.

### Enrichment analysis of differential metabolites in patients with and without cerebral infarction

**Figure 5:**
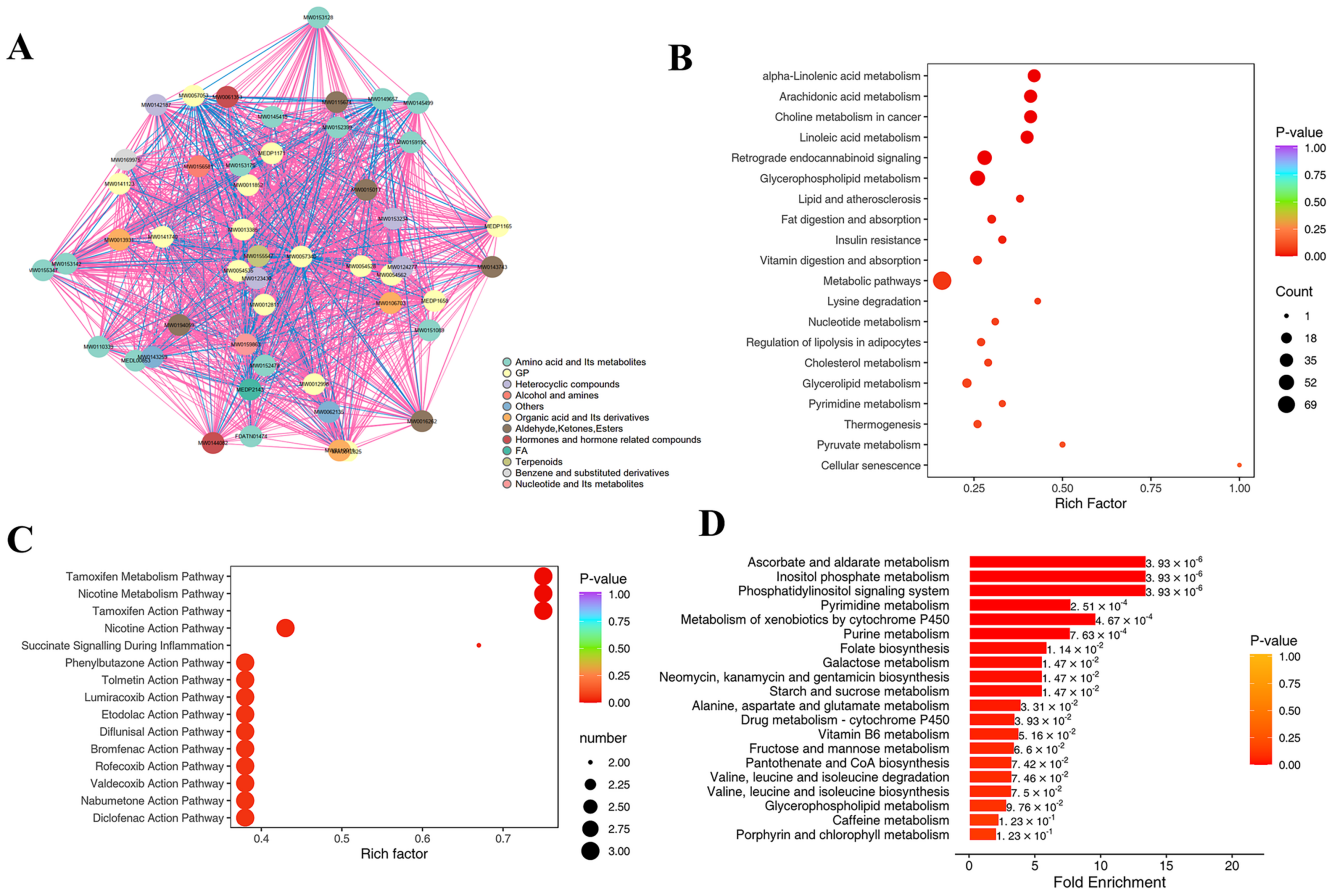
Enrichment analysis of differentially metabolites in the serum metabolome of patients with and without cerebral infarction. **(A)** Correlation network diagram of differential metabolites. The dots in the diagram represent different differential metabolites. Red lines represent positive correlations, and blue lines represent negative correlations. The thickness of the lines represents the absolute value of the Pearson correlation coefficient *r*. The thicker the line, the larger the |*r*|. **(B)** KEGG pathway enrichment analysis of differential metabolites. **(C)** HMDB functional enrichment analysis of differential metabolites. **(D)** MSEA pathway enrichment analysis of differential metabolites.

We further identified key metabolic signatures in patients with cerebral infarction compared with controls using metabolic networks and multidimensional enrichment analysis of the differentially expressed metabolites (Figure 5). Network analysis (Figure 5A) showed that the differentially expressed metabolites formed significant interaction clusters. There is a significant positive correlation between lipid metabolites (such as glycerophospholipids and cholesterol), while benzene derivatives and amino acid metabolites show a negative correlation. KEGG enrichment analysis (Figure 5B) indicated that these metabolites were primarily involved in lipid metabolism pathways (linoleic acid metabolism, arachidonic acid metabolism and glycerophospholipid metabolism), energy and inflammation-related pathways (retrograde endocannabinoid signaling and insulin resistance). HMDB analysis (Figure 5C) further revealed that these metabolites were closely associated with the metabolism of small molecules, including nonsteroidal anti-inflammatory drugs (NSAIDs), tamoxifen and nicotine. MSEA analysis (Figure 5D) revealed significant enrichment in pathways related to anti-inflammatory responses and tissue repair, including ascorbic acid metabolism and succinate signaling during inflammation. Collectively, these results indicate that patients with cerebral infarction undergo metabolic reprogramming characterized by disturbances in lipid, amino acid, and inflammatory metabolism, providing important insights into the underlying pathological mechanisms.

## Discussion

Cerebral infarction is a disorder of cerebral blood circulation caused by a variety of etiologies, resulting in ischemia, hypoxia and necrosis of brain tissue (25). Its primary pathological mechanism is blood flow interruption caused by cerebral vascular obstruction which poses an extremely high public health risk (26). In the treatment of cerebral infarction, the time window is crucial. Only by implementing intervention within a limited time after onset can patients achieve positive and effective treatment effects. However, due to time constraints, only approximately 3–5% of patients can receive these treatments in a timely manner and benefit. The limitation of treatment time directly affects the efficacy, resulting in approximately 70% of stroke survivors still having varying degrees of limb movement disorders or speech disorders, and even about 30% of patients being completely paralyzed (27). These outcomes severely compromise quality of life and impose substantial economic burdens on both families and society.

Metabolomics can be used to study the metabolic characteristics of brain tissue and blood to reveal disease-related metabolic disorders, and it is widely used in brain metabolism and cerebrovascular diseases (28). A metabolomics study on aneurysmal subarachnoid hemorrhage identified potential metabolite biomarkers that could be used for early diagnosis, predicting disease severity or complications, and guiding clinical intervention (28). This study compared the serum metabolic profiles of patients with and without cerebral infarction using metabolomics analysis. A total of 3,160 metabolites were identified. Benzene and its derivatives were found to be the most abundant in the serum of patients with cerebral infarction, while amino acids and their derivatives were the predominant components in patients without cerebral infarction. Previous studies have shown that aromatic compounds and their metabolites can induce oxidative stress and inflammation, exacerbating neuronal damage and cerebral endothelial dysfunction (29). A study on the serum metabolomics characteristics of 143 stroke patients and 59 controls at different time points found that phenylacetyl-L-glutamine levels increased over time (30). This is consistent with our study’s finding of a significant increase in aromatic compounds in stroke patients. In models of ischemic brain injury, benzene ring metabolites are closely associated with reactive oxygen species (ROS) production (31), and their accumulation may contribute to the development and progression of cerebral infarction by promoting platelet activation and thrombosis. The significant increase in benzene and its derivatives observed in this study reflects the increased levels of inflammation and oxidative damage in patients with cerebral infarction.

Amino acids are important precursors of neurotransmitters and are also involved in energy metabolism and cell repair (32). A previous study analyzing serum metabolism in 47 patients with cerebral infarction and 59 healthy controls found significant disturbances in amino acid, fatty acid and glycerophospholipid metabolism (33). This is consistent with the findings of this study. Studies have shown that glutamate, alanine and leucine levels are significantly downregulated in patients with acute cerebral infarction, which is closely associated with the post-ischemic energy crisis and neuronal damage (34). Insufficient amino acid supply leads to impaired glutamate-glutamine cycling which can affect neurotransmitter homeostasis and exacerbate neurological dysfunction. A GC–MS metabolomics study of acute ischemic stroke found significant increases in serum lactate and glutamate, while significant decreases in several amino acids and their derivatives, including alanine, serine, leucine and tryptophan (35).

In this study, fatty acid ratios were significantly reduced in patients with cerebral infarction, while glycerophospholipid levels were higher in patients without cerebral infarction. Abnormal lipid metabolism has been recognized as a key pathological basis for atherosclerosis and cerebral infarction. A study using metabolomics methods identified several differentially expressed metabolites in the serum of stroke patients, indicating that alterations in amino acid and fatty acid metabolism are closely related to ischemic stroke, such as L-tyrosine and lactic acid (36). Phospholipids are major components of cell membranes (37), and changes in their levels directly influence endothelial stability and inflammatory responses. Decreased free fatty acids reflect impaired energy metabolism and insufficient lipid mobilization (38). A metabolomics study based on UPLC/Q-TOF MS/MS identified uric acid, sphingosine, and adrenoyl ethanolamide as potential biomarkers of ischemic stroke (39). The study also identified significant changes in glycerophospholipid and sphingomyelin metabolic pathways, consistent with the findings of this study.

Differential metabolites were primarily concentrated in pathways such as linoleic acid metabolism, arachidonic acid metabolism, and glycerophospholipid metabolism. Studies have shown that arachidonic acid metabolites (such as prostaglandins and leukotrienes) mediate inflammatory responses and blood–brain barrier damage after ischemia (40) which can exacerbate neurological deficits. Disturbances in glycerophospholipid metabolism may affect cell membrane stability and signal transduction (41), impairing neuronal survival. Glycerophospholipid hydrolysis and monoacylglycerol turnover drive membrane turnover and provide substrates for arachidonic acid (AA) and its downstream prostaglandins/leukotrienes (42). The endocannabinoid 2-arachidonoylglycerol (2-AG) is released from monoglycerol lipase (MAGL) to generate prostaglandins in the brain, suggesting that the retrograde endocannabinoid signaling-AA metabolites-neuroinflammation axis plays a crucial role in the immune/glial response after cerebral ischemia.

This study found that phenylalanine and tryptophan-related pathways were significantly enriched. Evidence has shown that phenylalanine metabolites can affect central nervous system function by regulating neurotransmitter synthesis (43). Kynurenine (KYN) and its downstream metabolites in the tryptophan metabolic pathway are believed to be closely related to neuroinflammation and oxidative stress (44). In the acute phase when energy crisis and immune response are intertwined, the flow of amino acids is redistributed. Amino acids are mainly used for compensatory energy and antioxidant reactions and to drive the enhancement of the KYN pathway. Multiple studies have confirmed that multiple amino acids are downregulated in the acute phase of blood, and KYN metabolism is associated with stroke severity and prognosis (45).

This study further reveals the potential role of xenobiotic metabolism (such as the cytochrome P450 pathway) and anti-inflammatory/antioxidant pathways (such as ascorbic acid metabolism and succinate signaling) in cerebral infarction. This finding is consistent with previous studies. The CYP system is involved in the metabolism of drugs and xenobiotics, and it also metabolizes polyunsaturated fatty acids (PUFAs) to produce various oxidation products, such as epoxyeicosatrienoic acids (EETs) and hydroxyeicosatrienoic acids (HETEs). These metabolites regulate vascular tone, inflammatory responses and platelet aggregation (46). Furthermore, under ischemic stress, their excessive production can promote the accumulation of ROS and trigger a lipid peroxidation chain reaction (47). Previous studies have found that CYP-mediated lipid oxidation products are significantly elevated in the serum and brain tissue of stroke patients, directly contributing to the pathological processes of blood–brain barrier damage, neuronal necrosis and glial activation (48).

This study still has certain limitations. First, the sample size was relatively small, including only 20 participants, making it an exploratory and preliminary screening study. Although we identified significant metabolic changes associated with cerebral infarction, the small sample size may limit statistical power and the extrapolation of results. Second, this study has not yet validated the functional roles and potential molecular mechanisms of key metabolites using *in vitro* cell or animal models. Future studies should further validate these candidate metabolites in multicenter, large-sample cohort studies. Furthermore, by constructing cell and animal models, we should systematically evaluate the functional roles and regulatory mechanisms of these key metabolites in inflammatory responses, oxidative stress, and neuronal damage.

## Conclusion

This study comprehensively characterizes serum metabolic alterations associated with cerebral infarction. In patients with cerebral infarction, benzene derivatives and organic acids were upregulated, while amino acids and lipid metabolites were downregulated, indicating profound disturbances in aromatic amino acid metabolism, energy homeostasis, and lipid signaling. Pathway enrichment analysis revealed that linoleic acid metabolism, *α*-linolenic acid metabolism, retrograde endocannabinoid signaling, arachidonic acid metabolism, and glycerophospholipid metabolism are central to these metabolic disturbances, suggesting potential roles in inflammatory responses, neuronal injury and tissue recovery. Moreover, several differentially expressed metabolites were found to correlate with known metabolic alterations and physiological states, highlighting candidate biomarkers for early detection, prognostic assessment, and therapeutic intervention. This study provides a comprehensive view of the biochemical landscape in cerebral infarction, and lays a theoretical foundation for future clinical studies targeting metabolic pathways.

## Data Availability

The datasets presented in this study can be found in online repositories. The names of the repository/repositories and accession number(s) can be found at: https://www.ebi.ac.uk/metabolights/, MTBLS13031.

## Supplementary material

SUPPLEMENTARY FIGURE S1 Serum metabolomic analysis of patients without cerebral infarction. **(A)** Total ion current chromatograms (TIC) of serum from patients without cerebral infarction. **(B)** Analysis of metabolite class proportions in serum from patients without cerebral infarction.

SUPPLEMENTARY TABLE S1 Basic information of the study subjects.

SUPPLEMENTARY TABLE S2 Key putatively annotated differential metabolites identified in serum of cerebral infarction patients.
