# Supplementary figures and images for: LC–MS-based serum metabolomics reveals distinct metabolic signatures in patients with cerebral infarction
Source: Front Neurol. 2026 Apr 9;17:1714257. doi: 10.3389/fneur.2026.1714257 (PMC13102756; doi:10.3389/fneur.2026.1714257)

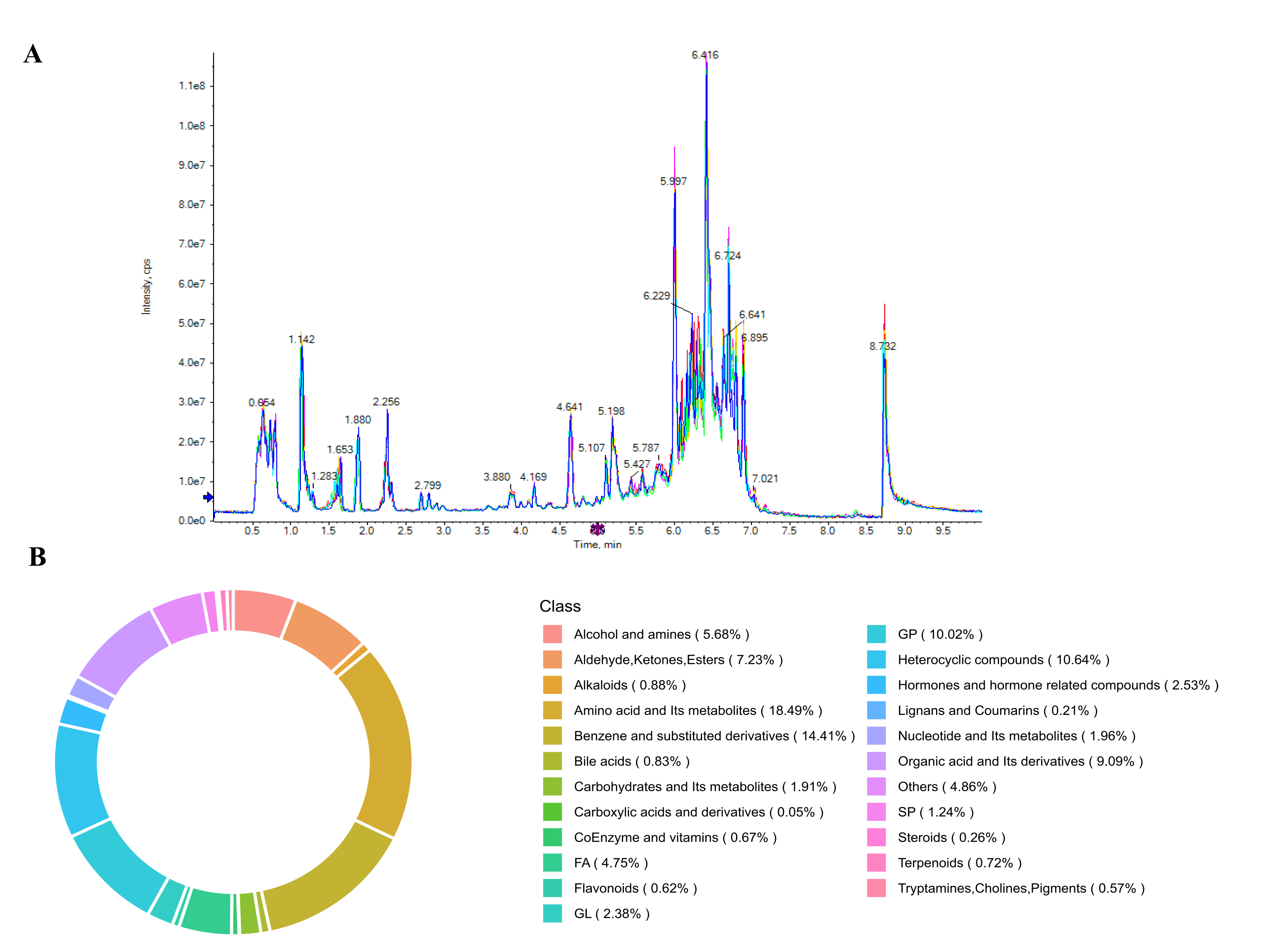

Supplement: SUPPLEMENTARY FIGURE S1 — Serum metabolomic analysis of patients without cerebral infarction. (A) Total ion current chromatograms (TIC) of serum from patients without cerebral infarction. (B) Analysis of metabolite class proportions in serum from patients without cerebral infarction. [file Image_1.tiff]
